# Tuning Electronic and Optical Properties of 2D/3D Interfaces of Hybrid Perovskites through Interfacial Charge Transfer: Prediction of Higher-Efficiency Interface Solar Cells Using Hybrid-DFT Methods

**DOI:** 10.1021/acsami.5c00201

**Published:** 2025-03-25

**Authors:** Hrishit Banerjee, Mohammad Khaja Nazeeruddin, Sudip Chakraborty

**Affiliations:** † School of Science and Engineering, University of Dundee, Nethergate, Dundee, Angus DD1 4HN, UK; ‡ Yusuf Hamied Department of Chemistry, University of Cambridge, Lensfield Road, Cambridge, Cambridgeshire CB2 1EW, U.K.; § School of Metallurgy and Materials, University of Birmingham, Edgbaston, Birmingham, West Midlands B15 2TT, U.K.; ∥ Group for Molecular Engineering of Functional Materials, Institute of Chemical Sciences and Engineering, École Polytechnique Fédérale de Lausanne, Lausanne 1016, Switzerland; ⊥ Materials Theory for Energy Scavenging (MATES) Lab, Department of Physics, Harish-Chandra Research Institute(HRI), A CI of Homi Bhabha National Institute (HBNI), Chhatnag Road, Jhunsi, Prayagraj 211019, India

**Keywords:** Perovskite solar cells, hybrid perovskites, quasi-2D, mixed-dimensional, bandgaps

## Abstract

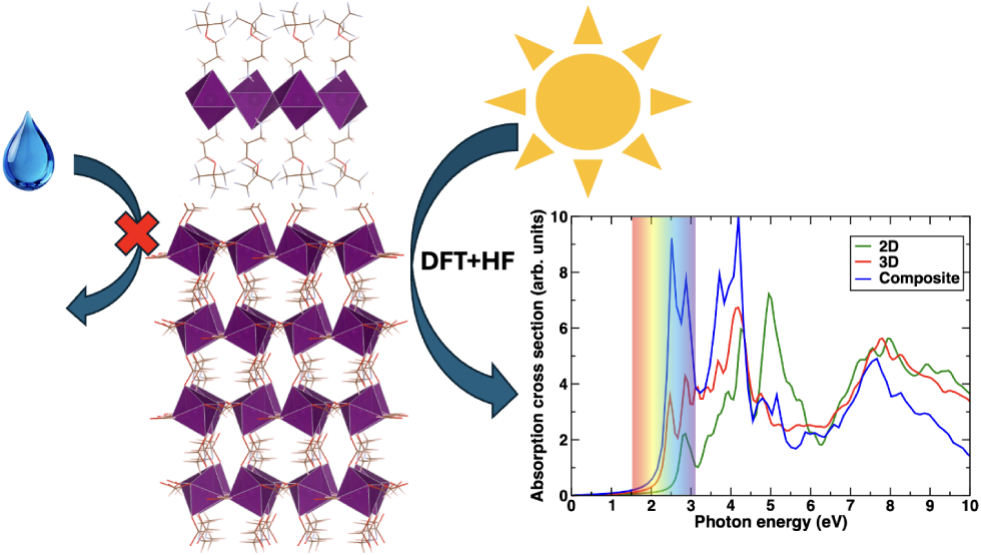

The 2D/3D or 2D/quasi-2D composite mixed-dimensional construction of hybrid perovskite interfaces is gaining increasing attention due to their enhanced stability toward degradation without compromising the corresponding solar cell efficiency. Much of this is due to the interfacial charge transfer and its consequences on the electronic and optical response of the composite system, which are instrumental in the context of stability and efficiency. In this work, we have considered a case study of an experimentally motivated 2D/quasi-2D interface constructed based on Ruddlesden–Popper phases of (A43)_2_PbI_4_ (2D phase) and (A43)_2_MAPb_2_I_7_ (quasi-2D phase) hybrid perovskites to envisage the unique tuning of electronic and optical properties through the associated charge transfer using density functional theory calculations based on both generalized gradient approximation as well as hybrid functionals, including corrections for nonlocal exchange obtained from Hartree–Fock. The corresponding tuning of the band gap is observed to be related to a unique charge transfer process between the 2D and quasi-2D counterparts of the interface mediated from the valence to conduction band edges of the composite. We have found that the optical absorption spectra can also be tuned by the construction of such a heterointerface and the emergence of a unique two-peak feature on the absorption edge, which is not present in either the 2D or quasi-2D hybrid perovskites. This feature is observed to be enhanced in the case of hybrid functionals, showing the importance of nonlocal exchange in optical spectra. We also compared the quasi-2D structure with the prototypical 3D structure MAPbI_3_ to show the progression of properties with dimensionality. The formation of the composite interface is found to increase the spectroscopic limited maximum efficiency for the use of these materials as solar cells from ≈24% for individual components to ≈32% for the composite heterostructure.

## Introduction

Perovskite solar cells (PSCs) have rapidly emerged as a promising alternative to traditional silicon-based solar cells due to their high efficiency and low cost.^1−6^ Hybrid perovskite solar cells have garnered immense interest in both experimental^7−9^ and theoretical condensed matter^10−13^ and materials research within the energy sector, owing to their extraordinary performance and ease of fabrication.^14−16^ Fundamental studies on PSCs are typically conducted through lab-scale procedures and carried out on small-area (≤1 cm^2^) devices. Commercialization of perovskite solar cells requires large-scale module manufacturing while maintaining a high performance. Spin coating succeeded in the fabrication of small-area devices with high performance, which is not suitable for uniform film formation during large-scale production. Several feasible large-area fabrication methods, including spray coating, blade coating, slot-die coating, and inkjet printing, have been proposed.^17^ Recently, various deposition methods, such as screen printing, slot-die coating, soft-cover coating, spraying coating, etc., have been developed to expand the device area from millimeters to hundreds of centimeters scale.^18^ The development of perovskite precursor inks suitable for use in low-temperature and vacuum-free solution-based deposition processes has also been reported.^19^

One significant challenge for PSCs is the aspect of short-term and long-term stability. The instability of PSCs is primarily influenced by environmental factors such as moisture and oxygen,^20,21^ thermal stress,^22^ and the intrinsic stability of methylammonium-based^23,24^ and formamidinium-based perovskites.^25^ Additionally, PSCs are susceptible to heating under applied voltage,^26^ photoinduced effects (ultraviolet light and visible light),^27^ and mechanical fragility.^28^ Several studies on PSC stability have been performed, and some elements have been proven to be important to the PSC’s stability.^29,30^ The water solubility of the organic constituent of the absorber material renders devices highly susceptible to rapid degradation in humid environments.^31^ The degradation induced by moisture can be mitigated by optimizing the constituent materials, the cell architecture, the interfaces, and the environmental conditions during the fabrication processes.^27^

Poor device stability due to degradation upon water exposure still impedes the widespread commercialization^5,32,33^ of hybrid perovskite solar cells. The effect of moisture on PSCs has received substantial attention on account of the presence of water under realistic operating conditions of solar cells.^34,35^ In the presence of moisture, hydrolysis of the perovskite occurs, which is triggered by the hygroscopic nature of the material. Prolonged exposure to water and high temperatures induces the deterioration of the solar cell electrodes due to reactions with the byproducts of perovskite decomposition. This phenomenon results in a drop in photovoltaic performance after just a few hours of operation.^36^

Among various perovskite materials, 2D/3D or 2D/quasi-2D architectures of hybrid perovskite interfaces have gained increasing attention in recent years for their enhanced stability and performance.^36−41^ Several seminal works have contributed extensively to the field, and it has been, for the last several years, a topic of great interest.^42−48^ These hybrid perovskites consist of both a 3D (or quasi-2D) perovskite structure and a 2D perovskite layer, which forms a protective barrier on top of the 3D/quasi-2D structure, leading to improved stability and reduced degradation over time.^37,40,49,50^ Several types of 2D perovskites like the Ruddlesden–Popper (RJ) phase, the Dion–Jacobson (DJ) phase, and the alternating cations in the interlayer (ACI) phase have been used.^51^ In these materials, it has been shown that interface energetics across 2D/3D or 2D/quasi-2D perovskite interfaces form a p–n junction that is capable of suppressing interfacial recombination losses, which has been a breakthrough in the field.^52^ One of the several proposed strategies to improve the stability that has recently emerged is the development of lower-dimensional (2D) perovskite structures derived from the Ruddlesden–Popper phases. The excellent stability under ambient conditions shown by 2D RP phase perovskites has made the scalability expectations burgeon since it is one of the most credible paths toward stable PSCs.^53^ Additionally, the 2D/3D or 2D/quasi-2D mixed-dimensional composite hybrid perovskite interfaces offer a unique band gap tuning capability, allowing for the absorption of a broader range of solar light and thus boosting the overall efficiency of the solar cell.^49^ When 2D or layered perovskites are combined with 3D or quasi-2D perovskites in a 2D/3D or 2D/quasi-2D mixed-dimensional composite hybrid, a synergistic action can be designed to boost efficiency and stability^54^ including high thermal stability.^40^ In particular, 2D/3D or 2D/quasi-2D composites, obtained by blending standard bulky organic cations (as an R component) with the precursor of the 3D perovskite or quasi-2D perovskite, have been recently embodied in solar cells to push device performance and stability. The main advantage of the mixed-dimensional composite over either 2D, 3D, or quasi-2D perovskites has been well-articulated by Wu et al.^55^ in their recent work, where they illustrate that 2D perovskites have high stability and low efficiency, while conventional 3D perovskites or quasi-2D perovskites have high efficiency but low stability. In contrast, the mixed-dimensional composite studied here achieves a balance of high efficiency and high stability.

In a recent work, the concept of engineering 2D/3D composites to create a low-dimensional perovskite (LDP) water-repellent sheath containing a saturated, highly fluorinated (fluorous) organic cation designed ad hoc on top of the 3D perovskite bulk was pushed further forward.^36^ The researchers evaluated the effect of the fluorous perovskite by incorporating the cation in two alternative ways: (a) by direct blending of the LDP and 3D perovskite precursors and (b) by engineering a controlled in situ layer-by-layer approach that enables the construction of a clean 3D/2D interface. They incorporated the LDP into two different 3D perovskite compositions, MA_0.9_FA_0.1_PbI_3_ (MFPI) and Cs_0.1_FA_0.74_MA_0.13_PbI_2.48_Br_0.39_ (CFMPIB). In the first case, the fluorous cation was added to the MFPI perovskite precursors, which may sometimes result in a mixture of 3D/2D or quasi-2*D*/2D perovskites in the bulk and on the surface. In the second case, the cation was deposited on top of preformed CFMPIB through a layer-by-layer passivation approach. In both cases, a thin layer of fluorous LDP self-assembled on the top surface of the 3D bulk or quasi-2D bulk, forming a waterproof sheath. The researchers envisaged the use of properly designed fluorous ammonium cations to modulate the dimensionality of perovskite materials and template the formation of LDP structures. Indeed, because of their shape and large size, much beyond one of standard MA and FA cations, which fit into the voids of the 3D or quasi-2D perovskite structure, these cations might act as effective spacers between PbX_6_ octahedra layers. The robustness of fluorous LDP compared to standard LDP might be enhanced due to the hydrophobic and solvophobic character of the perfluoroalkyl residues. Based on these premises, the researchers synthesized the fluorous cation (CF_3_)_3_CO(CH_2_)_3_ in the form of its iodide salt (named A43 from hereon). Two defined structures, arranged into the Ruddlesden–Popper phase of (A43)_2_PbI_4_ (*n* = 1) and (A43)_2_MAPb_2_I_7_ (*n* = 2), as derived by X-ray diffraction (XRD) measurements, were obtained. An excellent balance of efficiency and stability as per^55^ could hence be obtained with an *n* = 1 (2D) coating on an *n* = 2 (quasi-2D) perovskite^56−58^ as considered in this case. In the case of (A43)_2_PbI_4_ perovskite structure, bilayers of bulky A43 cations, measuring 20.65 Å in length, intercalate between monolayers of PbI_6_ octahedra. For (A43)_2_MAPb_2_I_7_, bilayers of A43 cations intercalate between bilayers of PbI_6_ octahedra, in which MA cations are confined, with the distance between the MA cations in two contiguous inorganic slabs being 26.64 *Å*. Thus, the researchers experimentally found a moisture-stable interface of 2D/quasi-2D hybrid perovskites. Although this interface has been studied experimentally, the microscopic properties of this interface from the point of view of electronic structure remain largely unexplored, which is the focus of this paper.

Hence, motivated by the success of this 2D/quasi-2D perovskite interface in overcoming moisture-based degradation observed experimentally, which makes this interface desirable for highly stable solar cell applications, in this work, we examine the electronic structure of this particularly interesting and experimentally designed hybrid perovskite interface. Using first-principles hybrid density-functional theory (DFT) calculations, the state-of-the-art approach for these massive interface systems, we investigate the basic electronic structure, charge transfer, and optical absorption properties of this experimentally designed 2D/quasi-2D hybrid perovskite interface and examine the impact of reduced dimensionality in the quasi-2D structure by comparing it with the 3D structure of MAPbI_3_. We find that there is definitive charge transfer occurring between the 2D and quasi-2D structures, which leads to a tunable optical band gap and tunable optical spectra. Our study concludes that the construction of a 2D/quasi-2D hybrid perovskite heterostructure results in enhanced tunability properties in terms of band gaps and optical absorption, as well as an increase in the maximum efficiency of solar cells.

## Computational Details

Our first-principles calculations were carried out in the plane-wave basis as implemented in the Vienna Ab-initio Simulation Package (VASP)^59,60^ with projector-augmented wave (PAW) potentials.^61^ As the exchange-correlation functional, we used the generalized gradient approximation (GGA) implemented following the Perdew–Burke–Ernzerhof prescription.^62^ For ionic relaxations, the internal positions of the atoms were relaxed until the forces became less than 0.005 eV/Å. This is a rather large composite structure and hence computationally expensive; however, in order to maintain the accuracy of our simulation results, we maintained a high cutoff of 500 eV throughout the calculations. A 4 × 4 × 2 Monkhorst–Pack *k*-point mesh was found to provide good convergence of the total energy in self-consistent calculations, which is found to be adequate considering the large size of the structure and its insulating nature. Benchmarking plots S1 and S2 are provided in Supporting Information.

All of the considered structures have been fully relaxed and converged to stable minimum energy configurations with the lowest ground-state energy using the conjugate gradient-type energy minimization technique. The DFT-D3 method with the Becke–Johnson damping function was considered for van der Waals dispersion correction.

Even though all the structures are fully relaxed, and there is no external unbalanced force/stress on the composite system, one can envisage a ″theoretical″ strain on the 2D and quasi-2D components in the composite supercell by virtue of the mismatch in corresponding lattice constants, even though there are no external forces on the lattice. The ″theoretical″ strain on the nD system due to lattice mismatch is calculated as the ratio of the difference in the a and b lattice parameters between the nD structure and the composite structure, expressed as a percentage. Thus, this is given by the formula:
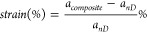
1

Charge analysis was carried out by the Bader charge partitioning method. Charge density difference calculations were performed using the formula:
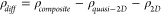
2

For this purpose, separate calculations are carried out by placing the 2D and quasi-2D structures within the composite geometry. The electron localization function (ELF), which indicates the probability of finding an electron in the neighborhood space of a reference electron located at a given point, is plotted for the composite to show the nature of the bonding in the system.

Hybrid functional calculations were carried out following the prescription of Heyd–Scuseria–Ernzerhof (HSE). The functional used in hybrid calculations can be mathematically expressed as in eq 3.
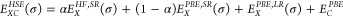
3which is the range-separated HSE functional, where α is the fraction of Fock exchange and σ is an adjustable parameter controlling the short-rangeness of the interaction. Here,  denotes the short-range Hartree–Fock (HF) exchange functional,  denotes the short-range PBE exchange functional,  indicates the long-range PBE exchange functional, and  refers to the correlation functional as given by PBE. The standard value of σ = 0.2 (referred to as HSE06), along with the standard value of α = 0.25, was used. HSE hybrid functionals, due to the presence of a fraction of Hartree–Fock exchange, have a nonlocal character and lead to a better description of the energies of nonlocal orbitals like p orbitals.

Optical absorption spectra have been determined at the level of the independent particle approximation from the frequency-dependent dielectric function given in eq 4:
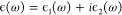
4They are evaluated by taking the average of the imaginary part of the dielectric constant.

″Spectroscopic limited maximum efficiency (SLME)” gives the highest theoretical efficiency of a material for use as a solar cell and is calculated à la Yu et al.^63^ by their method, which takes into account the band gap, the shape of the absorption spectra, and the material-dependent nonradiative recombination losses. The input for this is primarily direct and indirect band gaps determined from the band structure and the optical absorption coefficient. The optical absorption coefficient is calculated as in eq 5:
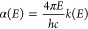
5where *k*(*E*) is the extinction coefficient and is given by eq 6:^64^
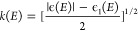
6

The absorption coefficient gives us the penetration depth of a photon with a particular energy before it can be absorbed by the material. The SLME (η) is defined as in eq 7:
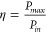
7where *P*_max_ is the maximum power density obtained and *P*_*in*_ is the incident power density of the solar spectrum. The *I*-*V* characteristic of the solar cell gives the maximum power density according to the formula as in eq 8:
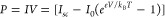
8where *I* and *V* are the total current density and potential over the absorber layer, respectively, *k*_B_ is Boltzmann’s constant, *T* is the temperature, and *e* is the elementary charge of an electron. *I*_*sc*_ and *I*_0_ are the short-circuit current density and reverse saturation current density, respectively. The absorption coefficient of the material α(*E*), the AM1.5G solar spectral function *A*_*sun*_(*E*), and the blackbody spectral function *A*_*bb*_(*E*,*T*) define the *I*_*sc*_ and *I*_0_ as in eqs 9 and 10:
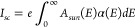
9
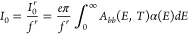
10

 is the radiative recombination current density, and *f*^*r*^ is the fraction of radiative recombination, which is defined by following eq 11:
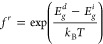
11where  and  are the indirect and direct band gaps, respectively.

## Results and Discussion

We have systematically determined the electronic structure, optical properties, and charge density distribution to envisage the possible charge transfer mechanism as obtained in the experimental investigations for the interface of 2D/quasi-2D hybrid perovskites: (A43)_2_PbI_4_ and (A43)_2_MAPb_2_I_7_. Both these structures are derived from experimental studies and are consequently obtained from X-ray diffraction, as explained in the introduction in ref.^36^ We have investigated the electronic structure of the individual systems, i.e., (A43)_2_PbI_4_ (2D phase) and (A43)_2_MAPb_2_I_7_ (quasi-2D phase), as well as the corresponding composite interface of mixed-dimensional character constructed between the quasi-2D and 2D nanostructures. It is to be noted that the *n* = 2 structure has a quasi-2D nature, and the connection in the z direction is due to organic ligands. For the sake of completeness and to understand the impact of the low dimensionality in the quasi-2D structure, we compare its electronic properties with a true 3D structure like MAPbI_3_. We describe the results obtained in this section.

### Crystal Structure Analysis

**Figure 1:**
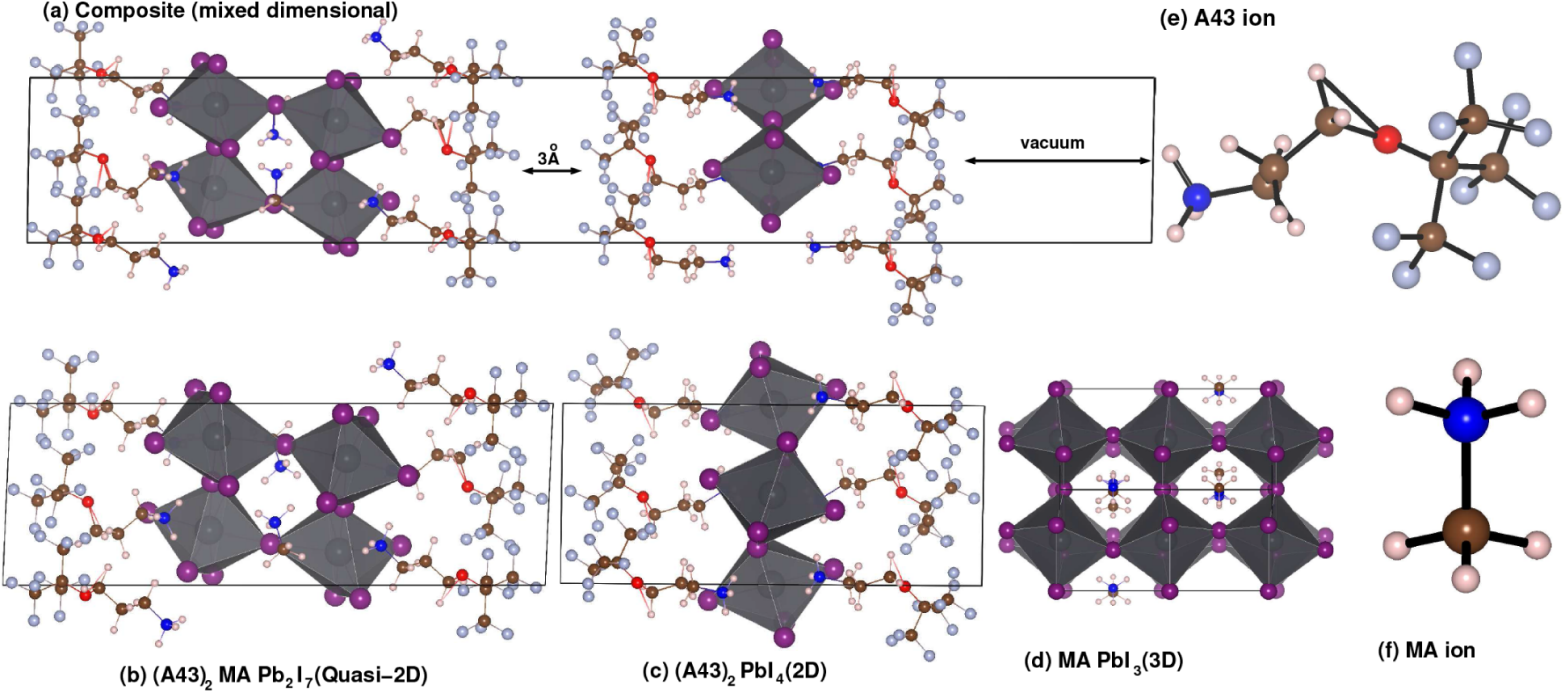
Crystal structures of (a) the mixed-dimensional composite structure with a typical 3 Å van der Waals gap in the top panel, (b) the quasi-2D perovskite, (c) the 2D perovskite, (d) the 3D perovskite, (e) the A43 ion, and (f) the MA ion. The respective PbI_6_ octahedra are marked in gray, and the atoms are marked with Pb (gray), I (purple), C (brown), N (blue), F (cyan), O (red), and H (pink).

We first present the crystal structure of the two hybrid perovskites and their composite structure, the prototypical 3D structure, and the individual ions, where the minimum energy and minimum force configurations of all three systems have been obtained from rigorous first-principles electronic structure calculations, as shown in Figure 1. The lattice constants of the 2D structure are *a* = 8.871 Å, *b* = 8.869 Å, *c* = 21.202 Å, *α* = 78.410 °, β = 89.161 °, γ = 88.252 °, and those of the quasi-2D structure are *a* = 8.808*Å*, *b* = 8.810*Å*, *c* = 26.637*Å*, α = 81.141_0_, β = 87.307 °, γ = 88.377 °. The lattice parameters of the supercell representing the interface are *a* = 8.856*Å*, *b* = 8.991*Å*, *c* = 68.569*Å*, α = 86.977 °, β = 87.498 °, γ = 88.194 °. The lattice parameters for the 3D MAPbI_3_ structures are *a* = 8.991*Å*, *b* = 13.123*Å*, *c* = 8.650*Å*, α = 90 °, β = 90 °, γ = 90 °. The individual structures, as well as the superlattices, were relaxed using DFT for both lattice constants and ionic positions to minimize any external unbalanced force or strain effects in the systems. As in experimental studies, the quasi-2D structure is considered as a substrate by fixing the coordinates of the base perovskite layer during geometry optimization, which is the standard procedure for mimicking a substrate effect^65−70^ on which the 2D structure is grown at a separation of roughly 3 Å between the two structures. This separation corresponds to a typical van der Waals-like distance and represents the optimum distance at which the two structures are stabilized. This has been confirmed after considering several other possible distances of separation between the two structures, and this was found to be the lowest energy composite structure. We have considered a vacuum of 20 Å on top of the 2D structure to nullify the interaction of the periodic images of the surface with the substrate along the z direction. The ″theoretical″ strain on the 2D system (in the *x*–*y* plane, since the z direction has vacuum) due to lattice mismatch is calculated as the ratio of the difference in the *a* and *b* lattice parameters between the 2D structure and the composite structure, expressed as a percentage. These values are −0.178% in the x direction and +1.36% in the y direction. Similarly, the strain on the quasi-2D lattice is calculated as 0.54% in the x direction and 2.01% in the y direction. We shall discuss any potential impact of these strains on band gap tuning in the section on electronic structure. It is important to note that the strain generated is due to lattice mismatch: the actual composite structure has no external unbalanced forces or stress and has been fully relaxed, as evidenced by the lattice constants, which differ from those of either the 2D or the quasi-2D structure. Hence, it is important to understand that the strain here is calculated by considering the difference in lattice parameters of the individual bulk systems; the structures themselves are not under any force. Additionally, it should be noted that these structures are massive, with 118 atoms in the 2D structure, 142 atoms in the quasi-2D structure, and 260 atoms in the composite interface structure, making the calculations extremely computationally and memory intensive.

### Electronic Structure Analysis

**Figure 2:**
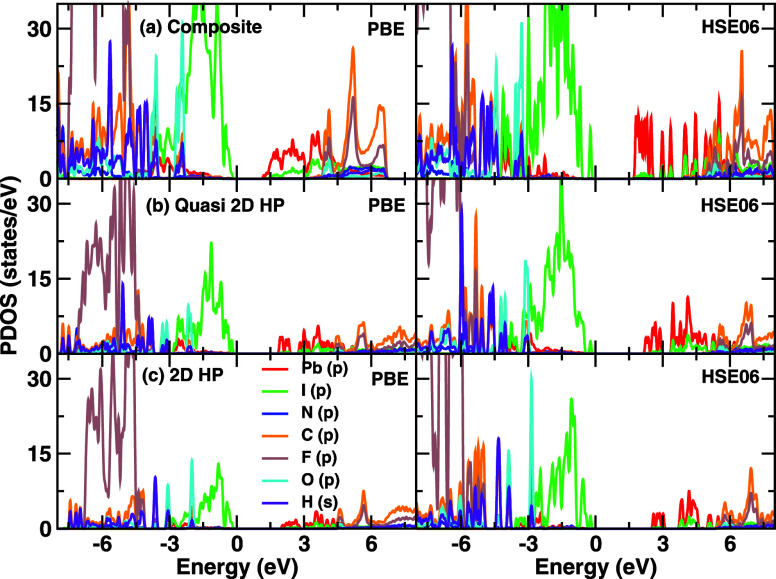
Partial DOS of the (a) composite, (b) the quasi-2D and (c) the 2D structures, respectively.

The projected density of states of the individual structures and the experimentally obtained composite interface structure are depicted in Figure 2, which primarily explains the electronic properties of the individual and composite structures for both PBE and HSE06 functionals. The left panel of Figure 2 shows the projected density of states (PDOS) from the PBE functional, and the right panel shows the PDOS obtained from the HSE06 functional. The PDOS is presented to elucidate the contribution of each element and their associated signatures in the valence band and conduction band regimes of the individual and composite structures. For the PBE functional in both the 2D and quasi-2D systems, the dominant contribution close to the Fermi energy arises from the I-p orbitals, with a small contribution from oxygen and other p-band elements. We have also observed the dominant contribution of iodine in the composite structure.

An interesting point to be noted from the projected DOS is that the band gaps in the case of the quasi-2D and the 2D structures are, respectively, ∼1.9 eV and ∼2.0 eV. However, in the composite structure, we obtain a band gap of ∼1.3 eV. This points toward a very illuminating finding: by constructing the composite structure of a 2D coating on top of the quasi-2D perovskite, the band gap changes, and this may serve as a method for tuning band gaps, which is an extremely important factor in the construction of perovskite solar cells. The change in the band gap in the composite structure compared to the quasi-2D structure may be influenced by the small amount of tensile strain in the composite structure compared with the quasi-2D structure. However, it is important to note that this is an indirect implication, as the decrease in the band gap may be due to the impact of the tensile strain on the quasi-2D section of the composite, given that there is no actual strain on the system due to the full relaxation of the composite lattice. The ″theoretical″ calculated strain arises only from the lattice mismatch between the quasi-2D and the composite structure. The valence band maxima in all three cases originate from I-p orbital; however, from the band curvature seen in Figure S2, it can be understood that this contribution comes from the quasi-2D structure. Similarly, the conduction band minima in all three cases originate from the Pb-p orbital, but from the band structure, it can be observed that this has a very strong similarity to the band structure of the pristine quasi-2D structure. This can also be confirmed by comparing the projected band structures shown in Figures S3–S5. It is, however, worth noting that this construction could form a mixed perovskite in the bulk with a thin film on top of a quasi-2D perovskite. A very similar distribution of PDOS is observed in the case of the HSE06 functional, albeit with slightly increased band gaps of ∼2.4 eV and ∼2.6 eV for the quasi-2D and 2D structures and ∼1.5 eV for the composite interface structure. However, although the contributions of the different elements in the PDOS are similar in the cases of PBE and HSE06, there is some shifting of weights observed in HSE06 compared to PBE and an overall stretching of the PDOS, which reflects the nonlocal character of the states. This is well-captured by HSE06 due to the nonlocal character of the Fock exchange included in hybrid functionals. Although the energies of p orbitals are better described by the nonlocal Fock exchange in hybrid functionals like HSE06, there is also a slight tendency of HSE06 functionals in their standard form to slightly overestimate band gaps compared to PBE.^67^ However, when considering optical absorption spectra, the nonlocal character and the higher accuracy in describing the energies of p orbitals in hybrid functionals are highly advantageous and more accurate than standard PBE.^71^ There is some difference in the spectral lineshapes of the DOS themselves, which may have a significant impact on the optical absorption spectra. Although experimental measurements of band gaps for these exact compounds are not available, band gap measurements in similar materials have yielded a range of values of 2.2–3 eV for 2D hybrid perovskites^72,73^ and values of 1.9–2.1 eV for quasi-2D hybrid perovskites,^56−58^ which are very similar to the band gaps obtained in our calculations.

It is to be noted here that, although optical absorption spectra and band gaps may be determined very accurately in experimental studies, small defects, dislocations, impurities, and lattice imperfections, especially in mechanically fragile hybrid perovskite compounds, can influence the measurement of optical absorption spectra and band gaps. However, the overall PDOS has very similar characteristics near and around the Fermi energy; one may carry out the discussion regarding charge transfer and occupancies. Hence, we shall discuss this further in the section where we examine optical absorption spectra, where this has greater importance.

### Charge Density Analysis

**Figure 3:**
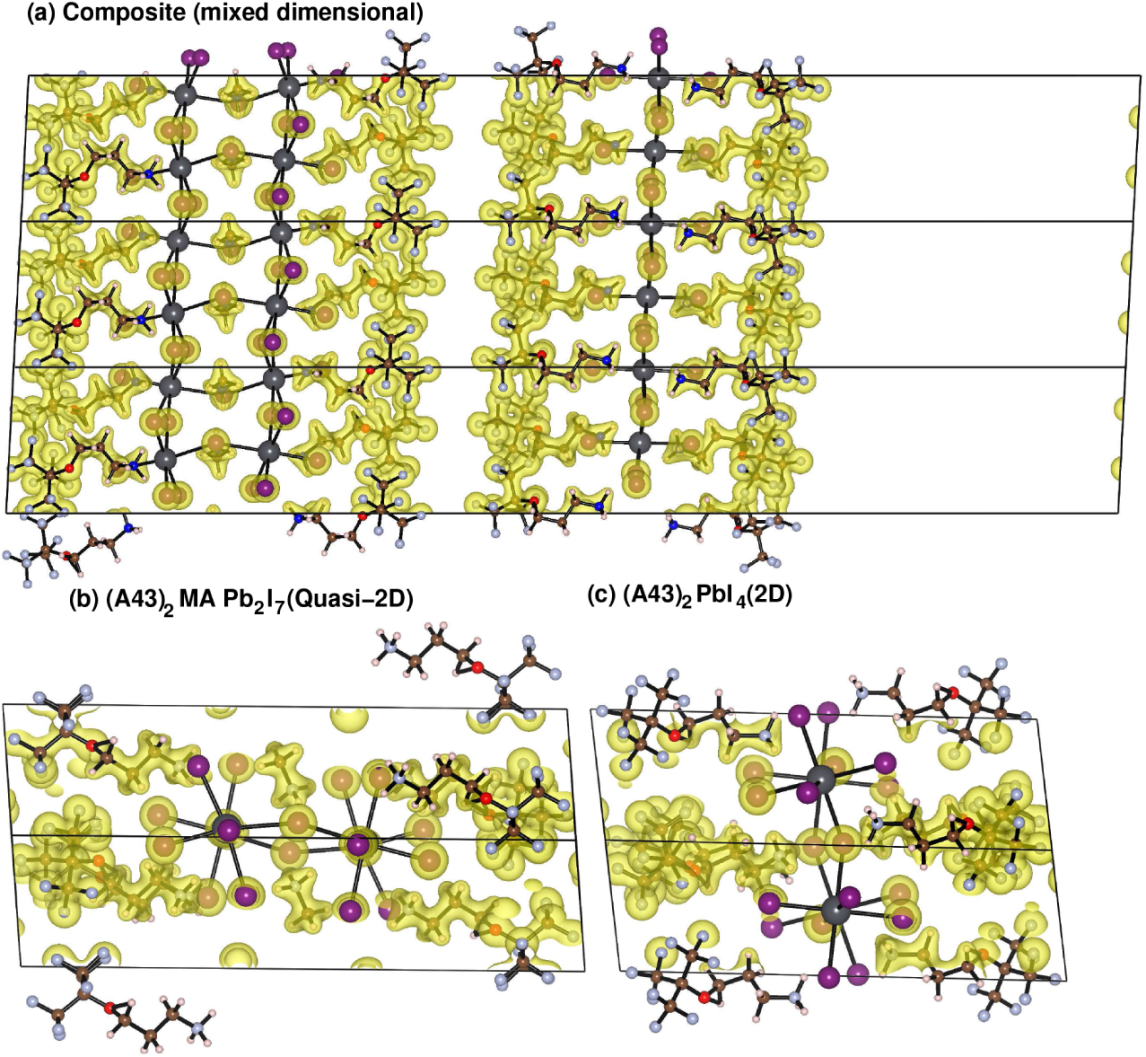
Charge densities of the (a) composite (mixed-dimensional), (b) quasi-2D, and (c) 2D structures in the bottom panel and the charge density of the composite in the top panel.

We can also relate the projected density of states to the associated ionic charges, as determined by performing a Bader charge analysis. Since the top of the valence band, as well as most of the other occupied bands in all the structures, exhibits very similar characteristics for both PBE and HSE06, we use the PBE functional here for the charge analysis, as HSE is extremely expensive and memory-intensive for such large structures. We have also visualized the charge density distribution of the pristine systems and the corresponding composite structure by calculating charge densities, as depicted in Figure 3. Comparing the overall charge per formula unit, one finds that while the 2D structure has 4.28 e/f.u. and the quasi-2D structure has 8.26 e/f.u., the composite structure exhibits an overall charge content of 12.28 e/f.u. It is evident that by bringing the structures together, there is a transfer of overall charge between the 2D and quasi-2D structures within their valence bands, which also leads to subsequent tunability of optical properties that depend on valence-to-conduction band transitions.

**Figure 4:**
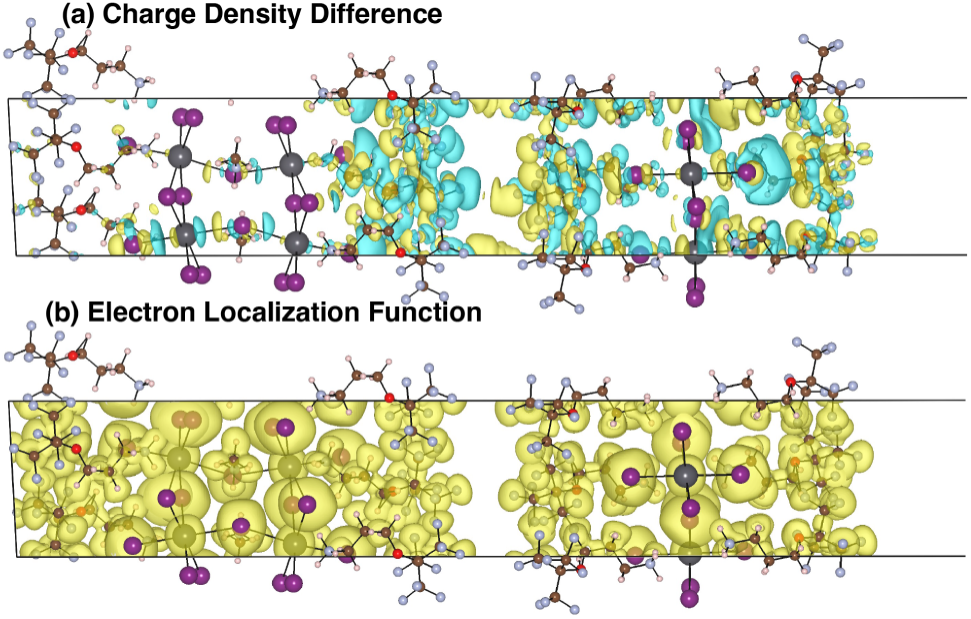
Charge density difference between the composite structure and the quasi-2D and 2D structures in the top panel and the electron localization function of the composite in the bottom panel.

Subsequently, to illustrate further where the charge transfer from the valence to the conduction band takes place and which species contributes the most to it, we explore the individual contributions of the dominating ions in the vicinity of the Fermi level, which is eventually iodine (I). We have found that in the 2D case, the contribution of the I-p charge is 2.74 e/atom, while in the case of the quasi-2D system, the associated charge is 2.77 e/atom. We also need to examine the individual charges in the composite structure from the associated I-p orbitals, respectively, with the 2D and quasi-2D structures. We have found that the iodine atoms from the quasi-2D part of the structure have a valence band contribution of 2.78 e/atom, while those from the 2D counterpart have a contribution of 2.88 e/atom. This shows two different things. First, we observe that I-p orbitals are electron-rich in the case of the composite compared to the individual 2D and quasi-2D structures. However, the 2D counterpart is more electron-rich than the quasi-2D counterpart in the composite structure. Due to the shorter Pb–I bond length of the 2D structure, there is higher hybridization between the Pb and I conduction orbitals. This eventually leads to a quasi-2D to 2D charge transfer mediated by a valence band to conduction band charge transfer. This idea is reinforced by the partial DOS, where the composite structure has more delocalized hybridized Pb–I conduction bands compared to the more localized conduction bands in the individual structures. A visual demonstration and further validation of the charge transfer phenomena are shown through the charge density difference (CDD) plot in Figure 4. This is calculated using the formula shown in eq 2 in the computational methods section. The yellow isosurfaces show positive charge transfer, and the cyan isosurfaces show negative charge transfer. Essentially, this shows where the charge has gone and from where in the individual structures compared to the composite. The very significant impact of the interface in the charge transfer phenomena is seen in Figure 4a where we observe that most of the charge transfer phenomena take place around the interface and the single 2D perovskite layer. There is very little charge transfer effect in the bulk of the quasi-2D perovskite. Hence, this demonstrates and validates the idea that the charge transfer phenomena are interfacial charge transfers. To demonstrate that this is different from bonding, we also show the bonding by plotting the electron localization function (ELF). This shows almost no impact near the interface, thus demonstrating the weak van der Waals nature of the bonding across the 2D/quasi-2D interface. Thus, there is clear evidence of the charge transfer that has occurred between (A43)_2_PbI_4_ and (A43)_2_MAPb_2_I_7_ mediated by a valence to conduction band charge transfer and a corresponding tuning of the band gap. Our theoretical study supports the experimental signatures of charge redistribution at the interface of quasi-2D and 2D structures during the interface reconstruction seen in existing experimental literature for similar 2D/quasi-2D interface systems.^74,75^ In both these articles, very similar charge transfer between 2D/3D or 2D/quasi-2D components of an interface system is demonstrated, and hence, our first-principles microscopic study provides an understanding of such charge transfer.

### Optical Absorption Spectra

**Figure 5:**
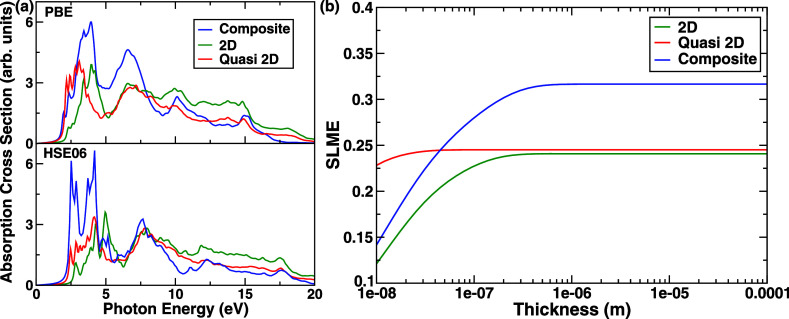
(a) Optical absorption spectra at the level of the independent particle approximation. A unique double peak feature is seen in the optical absorption of the composite material which is not seen in either of the bulk materials. (b) Spectroscopic limited maximum efficiency as a function of thickness calculated by the method prescribed by Yu et al.^63.^

Next, we examine the optical absorption spectra, as depicted in Figure 5a, of the individual structures and the corresponding composite system for both PBE and HSE06 functionals to envision the effect of heterostructure interface formation on the optical properties of the 2D and quasi-2D hybrid perovskites. This is extremely important, as the optical absorption spectra of any system under illumination which can be expressed as the variation of absorption cross-section as a function of photon energy, is one of the prime factors in defining the photoconversion efficiency of a solar cell.^76^

We employ both PBE and HSE06 functionals to describe the optical absorption spectra since first HSE is better suited to describe nonlocal exchange-based features in both DOS and optical spectra as well as the fact that some differences in the conduction band shapes were found in the DOS between the two different functionals. The nonlocal HSE functionals are usually more suited for the description of excited states in the calculation of theoretical spectroscopy and lead to more accurate results in terms of the match of spectral lineshapes with experimental spectra.^71,77^ This is not only due to the nonlocality of the Fock exchange but also due to the higher accuracy of the Fock exchange compared to PBE exchange in describing nonlocal p orbitals. Interestingly, if we observe the corresponding photon energy associated with the start of the absorption cross-section peak, we can see a clear change in going from the individual systems to the construction of the composite system for both functionals. One can relate this to the projected density of states of the considered systems, as the band gap of the individual system eventually governs the absorption peak in the optical spectra. The change in the band gap as discussed previously corresponds to the electronic structure of the composite system, which originated from the charge transfer between the 2D and quasi-2D hybrid perovskites mediated through the valence to conduction band charge transfer; therefore, the shift in the optical absorption spectra is well-connected to the charge transfer mechanism of the interface. The gaps are slightly larger in HSE06 compared to PBE, and hence, the absorption edge is shifted slightly to the right for HSE compared to PBE. However, there are a few more interesting things to be considered here. Let us consider the PBE spectra. First, the composite structure shows a double peak step feature at the absorption edge between 2.3 eV and 2.5 eV which is not seen in either of the individual absorption edges. A single peak step feature is seen in the 2D structure around 3 eV, but no such peak step feature is seen in the quasi-2D structure on the absorption edge. Thus, this feature on the absorption edge, which is the most important part of the optical absorption spectra in the context of solar cell-based application of perovskites, is completely new and forms due to the formation of the interface and can be attributed to the valence to conduction band charge transfer phenomena observed. This shows the tunability of the absorption edge, which is of prime importance for solar cell applications. This also points toward the tunability of the band gap and absorption cross-section by the construction of the 2D/quasi-2D interface which is a highly desirable property in solar cell materials. Although the higher energy absorption peak features in the composite mimic primarily the absorption peak features of the quasi-2D structure as expected, it is interesting to note that they are modulated by the presence of the 2D coating. It can be observed from Figure 5a that in terms of higher energy absorption features, the broad second peak in the quasi-2D bulk structures around 8 eV becomes much narrower and sharply defined, and now, there is a clear 2 peak feature in the absorption spectra of the composite structure; however, no such feature exists in case of the 2D or the quasi-2D structure individually, again reinforcing the idea of tunability of the absorption spectra by construction of the heterointerface. Next, we examine the HSE06 absorption spectra. We observe the double peak feature at slightly higher energies and a lot more pronounced, as well as the second set of two peak features seen in the composite IF structure. The first peak at 2.5 eV (495 nm) of the first doublet feature matches the quasi-2D absorption peak, and the second peak of the first doublet matches both the quasi-2D and the 2D absorption peak features. A dip after the first doublet corresponds to the dip in the 2D structure. Both the first and second peaks of the second doublet have contributory features from both quasi-2D and 2D absorption peak features as well, again pointing to the tunability of optical absorption. It is to be noted here that, although the measurement of optical absorption spectra is a standard procedure in hybrid perovskite-based solar cell materials, in these materials, which are mechanically fragile, small lattice defects or imperfections can influence the optical absorption spectra in terms of both the band gap and the lineshapes. Moreover, instrumental broadening can also smear out some of the features seen theoretically.

Next, we have calculated the spectroscopic limited maximum efficiency (SLME) based on the method proposed by Yu et al.,^63^ as shown in Figure 5b. This is derived directly from the absorbance data, which are obtained from the optical absorption data. As shown in Figure 5b, we find SLME values of 24.1% and 24.5% for the 2D and quasi-2D structures, respectively. For the composite structure, we find an SLME of 31.6%, which indicates an increase in efficiency compared to the component 2D and quasi-2D structures. It is important to note that although a theoretically significant increase in the maximum efficiency limit is predicted, the actual increase in efficiency of the coated composite might be smaller due to lattice imperfections like defects, impurities, dislocations, stacking faults, and surface imperfections which are common in hybrid perovskite systems.

### Comparative Analysis of Quasi-2D and 3D Structures

Although in this paper we primarily deal with an experimentally obtained 2D/quasi-2D interface, for the sake of completeness and to understand the impact of the lowering of dimensionality in the quasi-2D structure, we compare its electronic properties with those of a true 3D structure like MAPbI_3_. It is to be noted that, although currently 2D/quasi-2D interfaces with varying n for the quasi-2D case are generally considered an ideal geometry for desirable properties such as high efficiency and low moisture-based degradation, 2D/3D interfaces have also been studied extensively, as shown in the introduction. Hence, we compare the properties of a popular prototypical 3D hybrid perovskite with our quasi-2D (*n* = 2) structure.

**Figure 6:**
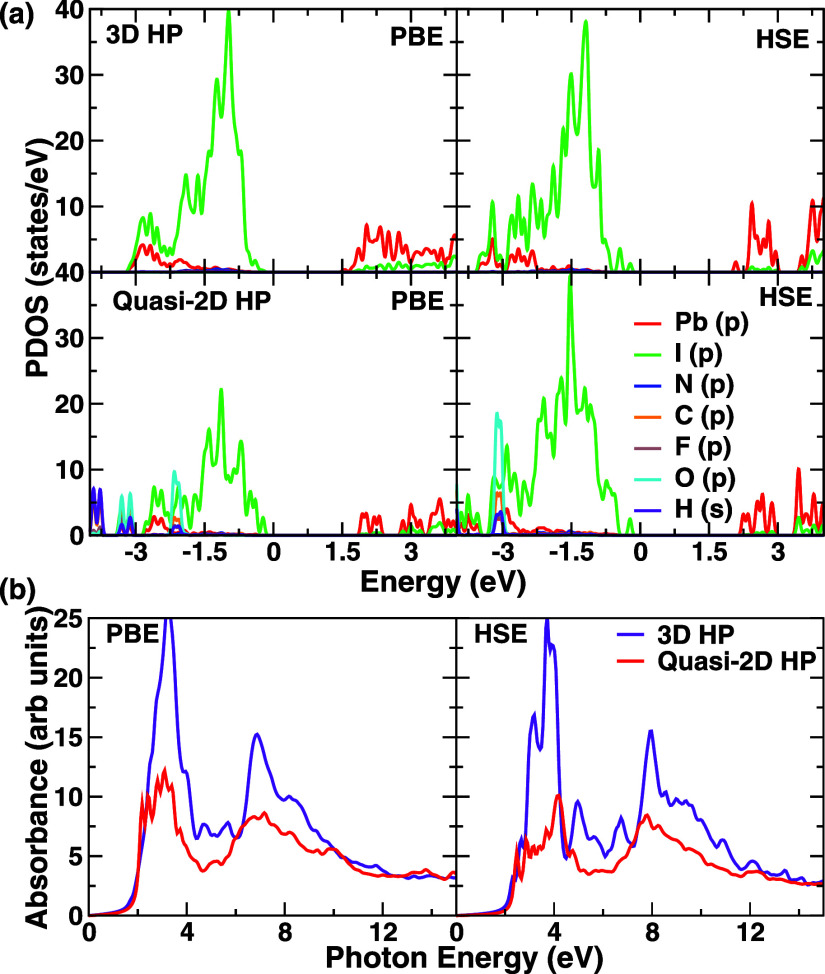
(a) The comparison of PBE and HSE PDOS for the quasi-2D and the 3D structures and (b) comparison of optical absorption spectra for these two structures for PBE and HSE functionals.

This provides a more detailed analysis of the electronic structure and helps to decouple the impact of the lower dimensionality of the quasi-2D structure from the actual charge transfer and consequent band gap tuning. The analysis of the electronic structure presented in Figure 6a shows very similar properties for the 3D MAPI structure and the quasi-2D (A43)_2_Pb_2_I_7_ structure. Both structures have valence band maxima populated by I-p orbitals and conduction band minima populated by Pb-p orbitals for both PBE and HSE functionals. The band gaps for the 3D MAPI structure are 1.5 eV (PBE) and 2.1 eV (HSE). The experimentally measured band gap of MAPI is ∼1.6 eV.^78^ This is comparable to the quasi-2D structure, as noted above, with band gaps of 1.9 eV (PBE) and 2.4 eV (HSE). This aligns with the slightly reduced band gap of the 3D structure compared to the quasi-2D and 2D structures. Hence, it is evident that since the quasi-2D and 3D structures have similar band structures and band gaps, it is also possible to use 3D hybrid perovskites as the core with a 2D hybrid perovskite coating on top.

Next, we compare the optical absorption spectra obtained using PBE and HSE functionals for the 3D HP and quasi-2D HP, as shown in Figure 6b. We observe that the absorption edge starts at very similar energies for both 3D HP and quasi-2D HP structures, albeit with slightly lower energies for 3D HP due to its lower band gaps. This indicates slightly higher efficiency for the 3D structure compared to the quasi-2D. The lineshapes at the PBE level are quite different; however, at the more accurate HSE level, with a better description of the nonlocal exchanges, a dual-peak feature appears in the 3D HP, which may be compared to the dual-peak nature of the composite. This may be attributed to several factors. The impact of charge transfer tuning the band gap and the composite being higher-dimensional than the 2D or quasi-2D structures both may play a role.

## Conclusion

In conclusion, in this work, we have envisaged the tuning of the electronic structure and optical properties of 2D/quasi-2D constructions of RP phases of hybrid perovskite through interfacial charge transfer. Considering the quasi-2D structure as the matrix and having a single layer of 2D hybrid perovskite constructed on top of the quasi-2D structure, we have found that the most stable heterostructure forms at a van der Waals distance of 3 Å between the quasi-2D and the 2D structures. From our electronic structure analysis, we have found that the majority of the contribution near the Fermi energy comes from I-5p orbitals hybridized with Pb-6p orbitals. We have also found that even though the 2D and quasi-2D structures individually have a band gap of around 2.4–2.6 eV, the composite structure has a band gap of 1.5 eV, which indicates that the band gap is modified by the formation of the heterostructure. We have performed a Bader charge analysis to explore the corresponding charge transfer mechanism at play, which eventually modifies the band gap. The charge transfer occurs from I-5p orbitals in the quasi-2D structure to the 2D structure, mediated by an inherent charge transfer from the valence to the conduction band and an eventual increase in Pb-6p–I-5p hybridization. We have found that the charge transfer mechanism has a profound impact on the optical absorption spectra, where a tunable absorption edge is observed with a novel two-peak step feature along the absorption edge that is not seen in either the 2D or quasi-2D hybrid perovskites. This is particularly important in terms of the tunability of optical absorption properties and applications of these 2D/quasi-2D composite mixed-dimensional hybrid perovskite heterointerfaces as solar cells. We also find an increase in the efficiency of the solar cell in the composite system compared to those of the individual 2D and quasi-2D systems. Our current investigation thus sheds light on the charge transfer phenomena between the 2D and quasi-2D hybrid perovskite systems when constructed in the interface form, which not only stabilizes the heterostructure but also governs the electronic structures in terms of the projected density of states and the tunable optical properties in terms of the absorption cross-section. We also compare the quasi-2D structure with a prototypical 3D hybrid perovskite and show that the electronic structures of the quasi-2D and 3D hybrid perovskites are similar, with similar band gaps; however, there exist small differences in the optical absorption spectral lineshapes, potentially due to differences in dimensionality. Our study is expected to give rise to further experimental studies examining the tunable optical absorption spectra in 2D/quasi-2D composite mixed-dimensional hybrid perovskite interfaces.

## Supporting Information Available

Convergence tests and bandstructures of 2D/3D hybrid perovskites (PDF)
