# Supplementary material for: Tuning Electronic and Optical Properties of 2D/3D Interfaces of Hybrid Perovskites through Interfacial Charge Transfer: Prediction of Higher-Efficiency Interface Solar Cells Using Hybrid-DFT Methods
Source: ACS Appl Mater Interfaces. 2025 Mar 25;17(13):19701–11. doi: 10.1021/acsami.5c00201 (PMC11969436; doi:10.1021/acsami.5c00201)
Supplement: Supplementary file 1 — am5c00201_si_001.pdf [file am5c00201_si_001.pdf]

Supporting Information: Tuning Electronic and Optical Properties of 2D/3D Interfaces of Hybrid Perovskites through Interfacial Charge Transfer: Prediction of Higher Efficiency Interface Solar Cells using Hybrid-DFT methods

Hrishit Banerjee,[*,†,‡,¶] Mohammad Khaja Nazeeruddin,[§] and Sudip Chakraborty[*,‖]

†*School of Science and Engineering, University of Dundee, Nethergate, Dundee, Angus, DD1 4HN, Scotland, UK.*

‡*Yusuf Hamied Department of Chemistry, University of Cambridge, Lensfield Road, Cambridge, Cambridgeshire, CB2 1EW, UK.*

¶*School of Metallurgy and Materials, University of Birmingham, Edgbaston, Birmingham, West Midlands, B15 2TT, UK.*

§*Group for Molecular Engineering of Functional Materials, Institute of Chemical Sciences and Engineering, École Polytechnique Fédérale de Lausanne, Lausanne, 1016, Switzerland.*

‖*Materials Theory for Energy Scavenging (MATES) Lab, Department of Physics, Harish-Chandra Research Institute(HRI), A CI of Homi Bhabha National Institute (HBNI), Chhatnag Road, Jhunsi, Prayagraj 211019, India*

E-mail: hb595@cam.ac.uk; sudiphys@gmail.com

Convergence tests

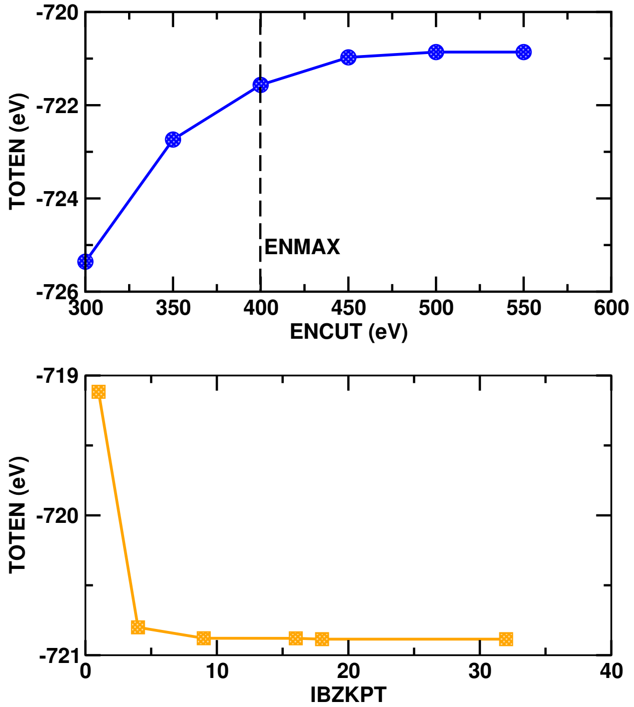

Figure S1: Total energy convergence for different Cut-off energies and Kpoint meshes.

Table S 1: KPOINTS grid and corresponding IBZKPT i.e. the number of KPOINTS. For these calculations, ENCUT was fixed at 500eV

KPOINTS	IBZKPT
$1 \times 1 \times 1$	1
$2 \times 2 \times 1$	4
$3 \times 3 \times 1$	9
$4 \times 4 \times 1$	16
$3 \times 3 \times 2$	18
$4 \times 4 \times 2$	32

Bandstructures of 2D/3D hybrid perovksites

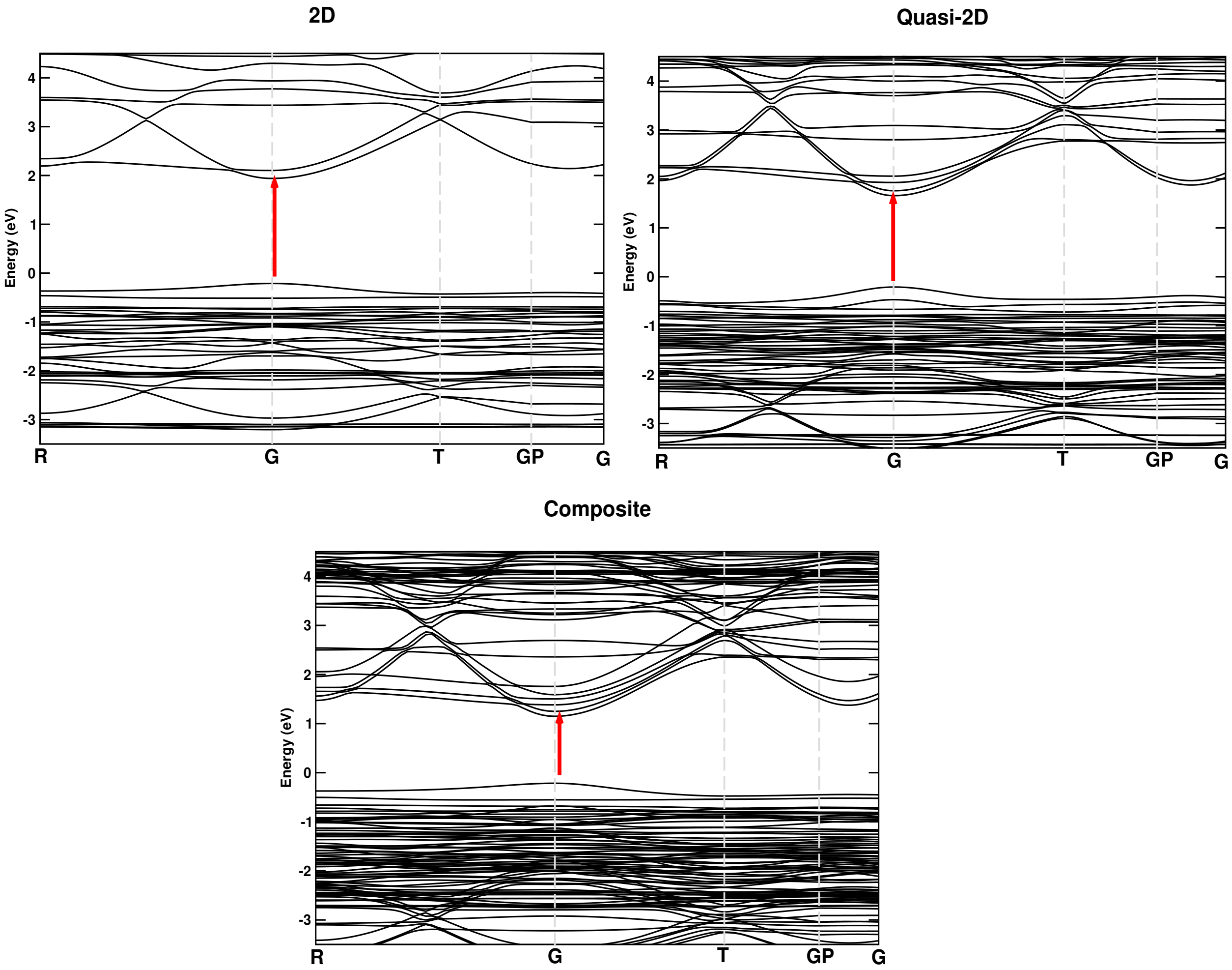

Figure S2: Total band structures for composite, quasi-2D and 2D materials. Band structure shows that the main optical transitions will occur from the valence band maxima to the conduction band minima which also corresponds to the direct band gap, marked by the red arrow.

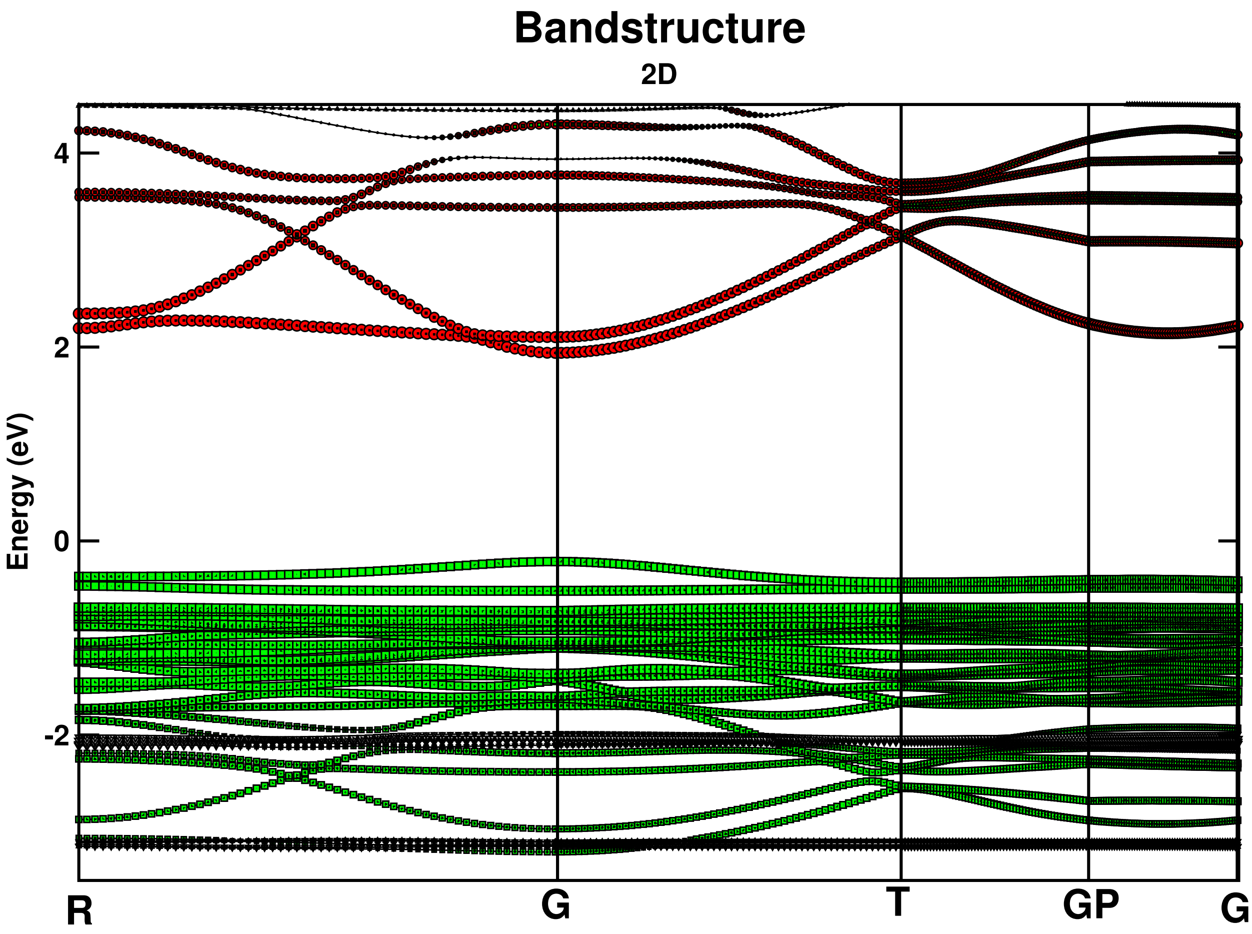

Figure S3: Projected band structures for the 2D structure. The colours correspond to the orbitals shown in Fig. 2. for PDOS, i.e. Pb-p (red), I-p (green) among the bands seen here.

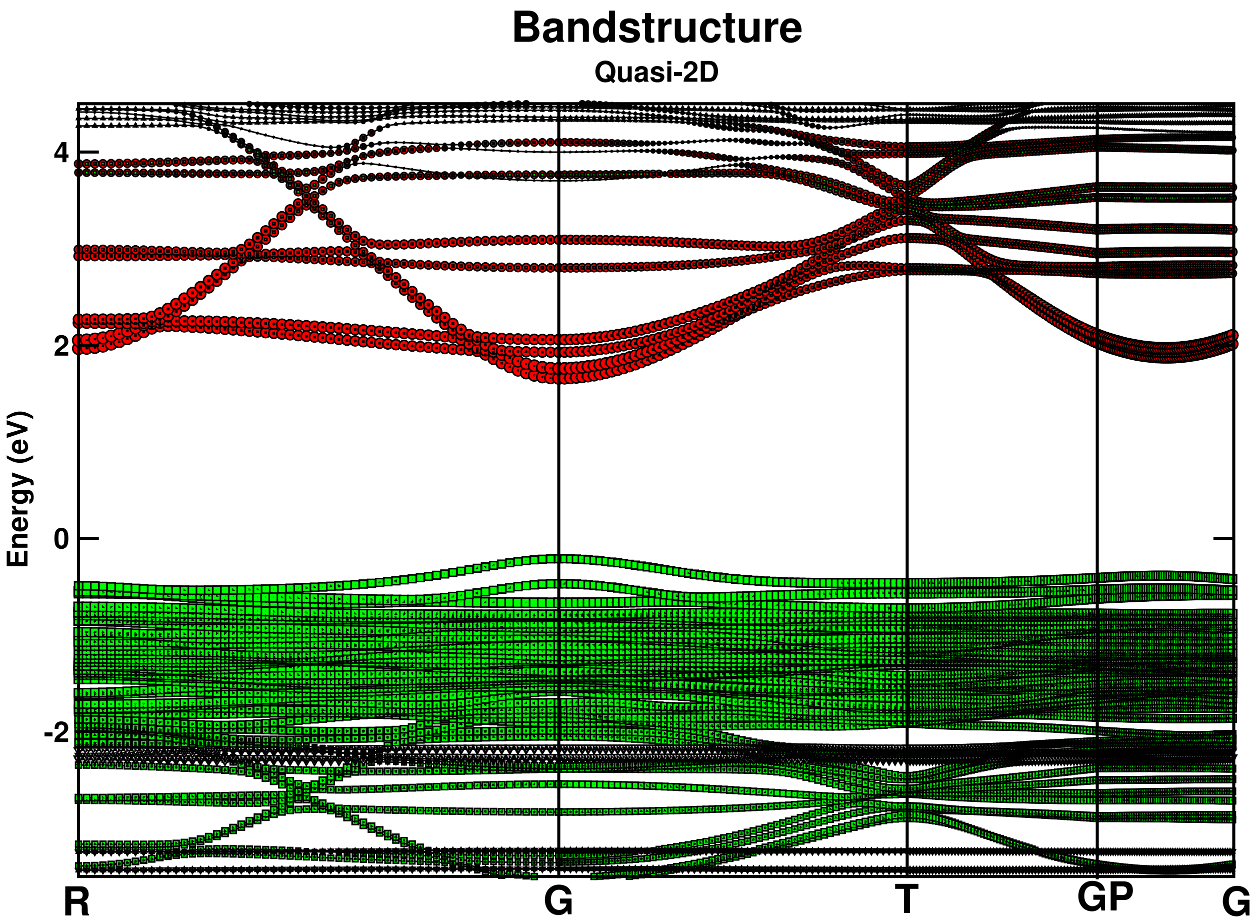

Figure S4: Projected band structures for the quasi-2D structure. The colours correspond to the orbitals shown in Fig. 2. for PDOS, i.e. Pb-p (red), I-p (green) among the bands seen here.

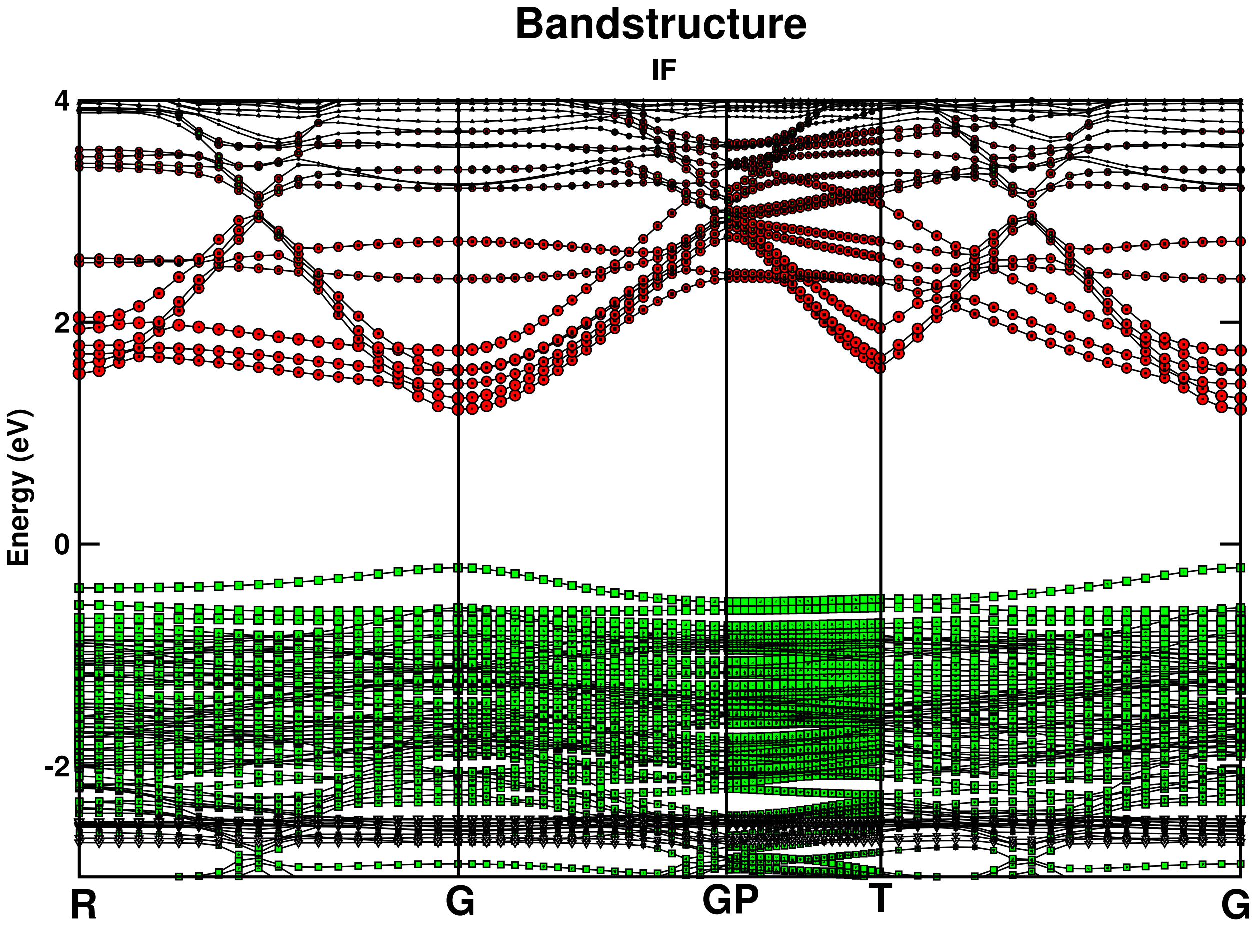

Figure S5: Projected band structures for the composite IF structure. The colours correspond to the orbitals shown in Fig. 2. for PDOS, i.e. Pb-p (red), I-p (green) among the bands seen here.
